# Impacts of COVID-19 and social isolation on academic staff and students at universities: a cross-sectional study

**DOI:** 10.1186/s12889-021-11040-z

**Published:** 2021-06-24

**Authors:** Walter Leal Filho, Tony Wall, Lez Rayman-Bacchus, Mark Mifsud, Diana J. Pritchard, Violeta Orlovic Lovren, Carla Farinha, Danijela S. Petrovic, Abdul-Lateef Balogun

**Affiliations:** 1 European School of Sustainability Science and Research, Hamburg University of Applied Sciences, Hamburg, Germany; 2 Department of Natural Sciences, Manchester Metropolitan University, Chester Street, Manchester, M1 5GD UK; 3 International Centre for Thriving, University of Chester, Chester, CH1 4BJ UK; 4 University of Winchester Business School, Winchester, SO22 5HT UK; 5 Centre for Environmental and Education Research, University of Malta, Msida, Malta; 6 Centre for Learning Excellence, University of Bedfordshire, Luton, UK; 7 Faculty of Philosophy, University of Belgrade, Cika Ljubina 18/20, Belgrade, 11 000 Serbia; 8 CENSE - Center for Environmental and Sustainability Research, School of Science and Technology, NOVA University Lisbon, Campus da Caparica, 2829-516 Caparica, Portugal; 9 Geospatial Analysis and Modelling (GAM) Research Laboratory, Universiti Teknologi PETRONAS (UTP), 32610, Seri Iskandar, Perak Malaysia

**Keywords:** University, COVID-19, Social isolation, Academic staff, Students

## Abstract

**Background:**

“The impacts of the Coronavirus Disease 2019 (COVID-19) pandemic and the shutdown it triggered at universities across the world, led to a great degree of social isolation among university staff and students. The aim of this study was to identify the perceived consequences of this on staff and their work and on students and their studies at universities.

**Method:**

The study used a variety of methods, which involved an on-line survey on the influences of social isolation using a non-probability sampling. More specifically, two techniques were used, namely a convenience sampling (i.e. involving members of the academic community, which are easy to reach by the study team), supported by a snow ball sampling (recruiting respondents among acquaintances of the participants). A total of 711 questionnaires from 41 countries were received. Descriptive statistics were deployed to analyse trends and to identify socio-demographic differences. Inferential statistics were used to assess significant differences among the geographical regions, work areas and other socio-demographic factors related to impacts of social isolation of university staff and students.

**Results:**

The study reveals that 90% of the respondents have been affected by the shutdown and unable to perform normal work or studies at their institution for between 1 week to 2 months. While 70% of the respondents perceive negative impacts of COVID 19 on their work or studies, more than 60% of them value the additional time that they have had indoors with families and others. .

**Conclusions:**

While the majority of the respondents agree that they suffered from the lack of social interaction and communication during the social distancing/isolation, there were significant differences in the reactions to the lockdowns between academic staff and students. There are also differences in the degree of influence of some of the problems, when compared across geographical regions. In addition to policy actions that may be deployed, further research on innovative methods of teaching and communication with students is needed in order to allow staff and students to better cope with social isolation in cases of new or recurring pandemics.

**Supplementary Information:**

The online version contains supplementary material available at 10.1186/s12889-021-11040-z.

## Background

During February and March 2020, following guidance from the World Health Organisation, governments around the world responded to the coronavirus pandemic by imposing restrictions on social contact. This affected almost all business sectors and public services, including the education sector [1, 2]. Once restrictions were announced, Higher Education institutions (HEIs) around the world found themselves in a new reality. According to global data for March 2020, schools and universities were closed for 87% of enrolled students and for more than 60 million teachers [3]. Due to concerns about the rapid spread of the virus, universities around the world very quickly postponed or cancelled all campus related activities, including teaching, lab-based research, examinations, sports, recreational and conference activities. These measures were taken to prevent or reduce the threat of the infection spreading at institutions in order to protect staff and students from the virus [2].

One consequence is that HEIs in many countries directed their teaching staff to move teaching and learning to online platforms - where possible - without delay, and to do so as comprehensively as possible [4, 5]. Since teaching and administrative staff and students in universities have variable levels of preparedness and experience in the use of online provisions, both groups have achieved diverse outcomes making the necessary transition to online education. For example, there have been difficulties using online platforms for examinations and for quality assurance and monitoring of students during tests and exams [6, 7]. Furthermore, practical assessments that require the use of laboratories or involve fieldwork have been unable to continue during this time [2], while some courses cannot be taught online [8].

More fundamentally, many HEIs, their staff and students, do not have the infrastructure to shift learning to online platforms immediately [2]. The implementation of online learning is expected to widen the learning gap between higher income and lower income families as has already been observed as the ‘digital divide’. Further, in developing countries the provision of online teaching and learning platforms is hampered because internet access and connectivity have imposed a limitation on access to education during the pandemic [9]. At the same time, some universities have had the relative luxury of adequate IT access and resources, to commence online support systems and counselling sessions to aid staff and students during these difficult times [2].

Apart from the hurried institutional responses to educational provision, there is emergent evidence that many individuals have struggled to cope with the multiple complications and consequences of lockdown Numerous international students were left stranded due to the travel restrictions, leaving some of them without accommodation or experiencing unexpected financial costs [10]. Similarly, unknown numbers of families will face unemployment and bankruptcy, that could make tuition fees unaffordable for some students, which itself will generate further anxiety [11]. Many academic staff found themselves working out of pocket, having previously paid for conferences and air tickets that became unusable because of travel bans [2, 12]. Many academic conferences quickly adapted, seeking to attract delegates to virtual platforms, such as the Academy of Management’s first Annual Meeting in 2020. While such on-line meetings may be less attractive as social and networking events compared with their physical cousins, they provide some value in the exceptional circumstances created by the pandemic [13].

Since the onset of Covid-19 and its ongoing prevalence and related lockdowns, HEIs around the world have assessed the financial impact as new - and continuing - student attendance on-campus and in residential accommodation seems increasingly unlikely. By example, Burki [14] reports that for the academic year 2019–20, the COVID-19 pandemic will have costed UK universities over £800 m, through lost income from accommodation, catering and conferences. In the USA, whereas the e HE sector earned about US$44·6bn in 2017; for 2019–20 the income is expected to have dropped to around $30bn. Similarly, Australia expects its HE sector to lose between AUS$3bn and $4·6bn in 2019–20.

Furthermore, the looming precipitous fall in tuition fees and accommodation charges from international students has exposed the financial viability of HEIs, especially those dependent on such income, typically in native English speaking and other developed economies. Given that staff salaries constitute at least 50% of HE institutional costs, leaders of UK and Australian universities are exploring financial survival options, including voluntary and involuntary redundancies, pay cuts and freezes, and abandonment of national pay guarantees, in the face of resistance from employee unions [15–17]. In a monthly survey of assessments of US college presidents of their most urgent concerns, the top three worries in May and June (2020) were ‘summer or fall enrolment’, ‘deciding fall term plans’, and ‘long-term financial viability of the institution’. The next most pressing issues were ‘mental health of students’ (4th at 33%) up from 6th (32%) in May, and ‘furloughing or reducing salaries for faculty and/or staff’ (5th at 31%). The same survey revealed m ental health of faculty and staff’ to be lower priorities, at the 8th position (26% in June, 19% in May) [18].

## COVID-19, shutdown, and social isolation of academic staff and students

Following the categorisation by the WHO of COVID-19 as a pandemic (11 February 2020), public health experts and authorities recommend *social isolation* as a primary measure to mitigate the spread of the SARS-CoV-2. People of all ages, including university students and academic staff in the majority of countries, were asked *to avoid physical social contact* and participation in group and community activities, family gatherings and public events. With few exceptions, self-isolation was suddenly required by nation states, particularly of individuals returning from more severely affected regions, as well as for older people and those with underlying health conditions. While self-isolation has been generally considered an act of individual responsibility, some countries introduced and enforced new specific regulation to restrict movement outside the home and to require the wearing of face masks, and established the authority to impose fines or imprisonment for non-compliance (DW Akademie, 2020).

Humans are fundamentally a social species: it is in their nature to interact and form various types of relationships with others. Social isolation has been understood as both an objective phenomenon experienced by individuals, such as that characterised by a ‘lack of social interaction’ [19], ‘the actual lack of social ties’ [20], and ‘social disconnectedness’ [21]**.** It is also understood as a subjective experience by individuals, such as a ‘lack of engagement with others’ [20], ‘loneliness’ [22] or ‘the perceived discrepancy between actual and desired social relationships’ [23]. The widespread mandated household confinement and mobility restrictions can be understood as creating *objectively* real physical isolation, immediately and severely reducing direct social interaction and contact with anyone outside the household. At the same time, these conditions create circumstances in which individuals *subjectively* experience social isolation.

Extensive evidence from social science and public health studies suggest that social interaction and relationships are important for mental wellbeing throughout the lifespan. For example, Hartup and Stevens [24] conclude that within the lifespan, friendships can foster a sense of wellbeing and self-esteem. Similarly, in their review of scientific studies, Umberson and Montez [25] conclude that over the lifespan, social relations do influence health and by extension, as others indicate, social isolation contributes to anxiety and depression ([26–28] [29]). Elsewhere, correlations have been found between the perceived lack of social connections and feelings of loneliness, with higher rates of morbidity and mortality [30], as well as of infection and cognitive decline (Cohen et al. 1997; Pressman et al. 2005; Barnes et al. 2004; Wilson et al. 2007, cited in [21]).

Similarly, strong correlations have been demonstrated between social relationships and physical health, such that more socially connected adults are found to be healthier and to live longer than their more isolated peers [25]. In addition, there is a link between being socially engaged and the experience of stress, although this link is complex; stress might be both a cause and effect of social isolation [31]. Furthermore, more socially engaged individuals seem to possess a relatively larger repertoire of restorative or stress-buffering resources (both behavioural and interpersonal) [31]. Studies focusing on relations between marriage/family status and perceived isolation reveal that there are lower levels of loneliness amongst individuals who are married (Hawkley et al. 2005; Pinquart and Sőrensen, 2003, cited in [22]), while married men gain greater health benefits than married women (Waite 1995, according to [25]).

Two decades before the COVID 19 pandemic, Killen (1998) observed an ‘epidemic of loneliness’ (according to [25]), partially linked to an increase of single-person households as observed in some countries, and which could account for a higher risk of actual or perceived social isolation. The unfolding of the pandemic crisis reveals further complexity in the nature of social isolation, in light of reports that the isolation of families was accompanied by increased domestic violence and online child abuse [3]. In addition, the level of educational attainment also shapes the extent to which individuals experience isolation. For example, those with higher education levels are found to develop more diverse social networking groups, which is associated with better mental health outcomes (Fiori et al., 2006; Li & Zhang, 2015; Litwin, 2001; Park et al., 2017; Windsor, Rioseco, Fiori, Curtis, & Booth, 2016, cited in [32]). Likewise, lower level of loneliness have been reported to be associated with the rise of educational level [22].

Research also suggests that age may be a factor influencing the level of health risks. Nonetheless, subjective perceptions of social support or isolation also play their role. Specifically, there is a tendency for young people to feel lonely even when surrounded by others or when being a member of a group of peers, while by contrast, the elderly might not feel lonely even when their social network is significantly reduced [19]**.**

Similar findings appear in studies on relations between age and social media use. There is some evidence that social media use may help people feel less isolated, such as through drawing support from online social networks such as Facebook, Instagram, and others; Hajek and König [23] found that adults over 40 years of age, as daily users of online social networks, ‘tend to feel less socially isolated than less frequent users or non-users’. Other researchers have found contradictory evidence. A study of adults in the US, aged 19 to 32 years, found linear associations between increased social media use and an increase in perceived social isolation [20]. This suggests that there is no such simple correlation between social media use and social isolation; age seems to matter.

These studies show that the factors influencing or otherwise associated with social isolation as a subjective experience are interdependent and complex, but carry consequences for morbidity and mortality outcomes. The studies also highlight that the experience of social isolation is context dependent and is at least a product of psychological and social factors. Influencing factors include health, whether or not the individual is in a close social relationship and the nature of that relationship, educational level and social networks, and age, among others. From a ‘human ecology theory’ perspective (e.g. [33]), these interdependencies ‘emphasise the fluid[ity] of relationship formation based on current environmental constraints’ ([20]: 6). Seen through the lens of social networks, and in particular the ‘social convoy’ theory [32, 34], individual lifespans traverse concentric social networks representing varying degrees of closeness, all dynamically shaping the course of an individual’s lifetime. From this view, the structure, function and quality of the social convoy reflects and shapes how each individual navigates between social integration and social isolation.

It should be noted that even without the social confinement imposed as a result of the COVID-19 shutdown, an association between working conditions and wellbeing was recognised and continues to be debated [35]. So too is workplace wellbeing also shaped by extra-organisational influences, such as family tensions to economic conditions [36].

Changing contemporary work patterns have been shown to affect social wellbeing [37]. Set against the premise that ‘high performance work systems’ or ‘high commitment workplaces’ (involving a great deal of employee discretion, autonomy and flexibility) develop intrinsic staff motivation, Boreham et al. [37] find significant adverse impacts of such contemporary work practices on social wellbeing. They find that the boundary between work and social wellbeing is blurred, with interpenetrating links between workload pressure and stress and impacts on quality of life.

Academic staff will be familiar with these stresses and strains, balancing their high commitment to their profession and identify within a stressful working condition with the need to attend to life outside the academy [38, 39]. In their study, Kinman and Wray [38] reported a trend of increasing stress among academic staff, running at a significantly higher level than other UK occupations. Students, especially post-graduate students, are prone to stress, anxiety and depression [40, 41], with which universities are familiar (e.g. Institute for Academic Development, ‘Preparing for Change’, University of Edinburgh) and for which much online advice exists (e.g. [42]), alongside other online HE policy research and resources (e.g. [43]). Regardless of any widespread appreciation of the links between wellbeing at work and social wellbeing, Cottini and Lucifora’s [44] study of 15 European countries highlighted the adverse effects of working conditions on mental health, due in large part to cross country differences in labour market flexibility, variations in their health and safety regulatory environments.

It seems likely that the overriding importance of an immediate implementation of the social lockdown will foreground its associated stressors, overshadowing the longer standing work stressors. As the lockdown persists, its stressors will compound existing work stressors and add new ones.

Set against this background, the research presented here aims to identify the impact of the COVID-19 lockdown on working conditions and on the social isolation imposed on academic staff and students of HEIs around the world. This study contributes insights to the subjective experiences of staff and students working in HEIs around the world, shining a light on their subjective constructs (i.e., the perceived level of institutional support or isolation) as a response to the enforced isolation. In addition, this study contributes by highlighting far reaching policy implications for teaching and learning approaches in the emerging context of the increased reliance on social interaction in an online environment - in particular, the need to secure technological enfranchisement of all students and the wellbeing of both staff and students. This study is paralleled by a research focusing on students’ mental health problems before, during, and after COVID-19 lockdown undertaken in Italy, with a sample of 358 Italian students aged 18–30 [45].

## Methods

Given the aim of this study, a cross-sectional survey research design was adopted to examine the experiences of academic staff and students in HEIs around the world. The design of the survey was informed by the literature on the impact of influences on work practices, and the influence of changing work practices on social isolation and wellbeing. Since the study into the links between COVID-19 lockdown and social isolation is time sensitive, a convenience sampling was appropriate, as it facilitates the timely gathering of data. It was also appropriate under the circumstances to gain responses from individuals at a time when, under the prevailing emergency circumstances, staff and students had other immediate concerns.

This non-probability sampling method also involves a combination of purposive and homogeneous methods, and respondent self-selection (Saunders et al., 2003). This strategy directly addresses respondents with experience of the questions raised in the survey and the ability to generate new insight. The online survey was conducted from 14th April to 4th May 2020 using Survey Monkey. It was initiated by the Hamburg University of Applied Sciences and disseminated via email through a web-link to the networks of the co-authors plus various mailing lists (e.g. LISTSERV). on teaching and research in higher education, thereby reaching students and academic staff across the globe. The survey link was also disseminated by email through the national and international personal and professional networks of the research team, defining this as a snowball method. Table 1 gives an overview of the research methods used for data collection, whereas Table 2 presents the methods used for data processing.

**Table 1:** Summary of the research methods used for data collection and their relations

Method	Usefulness	Relation to other methods
On-line survey	Gathering of data from a wide audience in many countries	Provision of the information to be statistically processed
Non-probability sampling method I (convenience sampling)	Quick and extensive reach to research networks, especially when participants are under crisis situation	Rapid access to initial sample
Non-probability sampling method II (snow ball)	Extend sample to relevant participants within sample networks	Extend sample within networks
Dissemination via web links, networks and mailing lists sampling	Extend sample to relevant participants beyond sample networks	Extend sample beyond sample

**Table 2:** Summary of the research methods used for data processing and their relations

Method	Usefulness	Relation to the others
Descriptive statistics	Provision of the information to be statistically processed on social-demographic issues	Analysis of trends and identification of socio-demographic differences
Inferential statistics	Provision of the information to be statistically processed on geographical distribution of responses	Assess significant differences among the geographical regions

As a result of the promotion efforts and after two reminders, a total of 711 questionnaires from 41^1^ countries were received. Even though this represents a wide distribution of respondents, it is unevenly distributed between geographic regions (with predominance from Europe) and scientific disciplines, and reflects a recognised implication of the convenience sampling strategy. The study cannot claim to be global, since not all parts of the world, such as Asia and Africa, are represented..

**Fig. 1:**
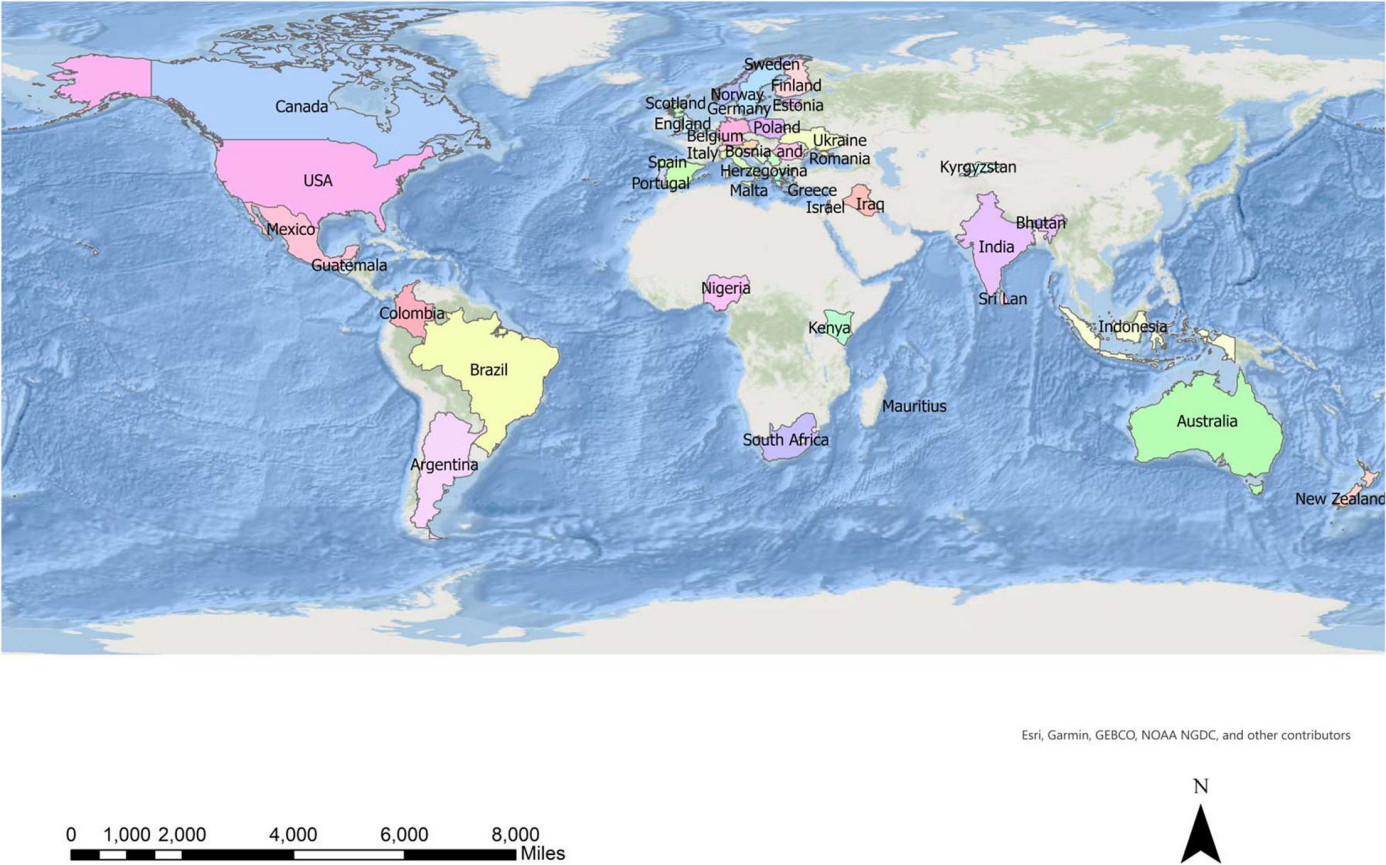
Countries in which respondents took part in the survey

Figure 1 shows a spatial distribution of the respondents’ countries.

The instrument was designed to gather (1) socio-demographic information about the respondents, including their role in the university, area of work, age and gender, with whom they live, and where they live (Q1-Q6, Q17, and Q18), and (2) perceptions of the extent to which the shutdown affected their capacity to perform their tasks as normal, in work or study (Q7-Q15). This included questions about the extent to which they felt that their institutions took adequate measures to help them perform effectively while in social isolation, namely through the (a) provision of necessary communications infrastructure and the (b) provision of other support for work/study from home. (Appendix,Table 3: Summary of questionnaire design).

A five-point Likert Scale was used with all questions. All the sections, subsections, and questions were given the same weight for scoring as the focus of the questionnaire was at the question level.

The instrument was also reviewed and revised through several iterations by all the individuals in the research team, which includes social science experts, and who themselves, in their distinct geographical contexts and as HE practitioners, were also experiencing the impacts of COVID and were able to interpret the validity of the questions. This process also ensured coherence and clarity and resulted in the removal of redundant questions. The survey was piloted by a panel of 10 experts in the areas of sustainability and education at different universities to ensure the reliability and validity of the responses. The complete questionnaire comprised 29 questions, but this study reports on parts one and two of the study (Q.1-Q.22). (Questionnaire provided as supplementary file).

Statistical analysis was performed through the Statistical Package for the Social Sciences version 25 (SPSS). Descriptive statistics were analysed to establish trends and identify socio-demographic differences. Inferential statistics (t-test for difference between groups and Analysis of variance - ANOVA) were used to assess significant differences among the geographical regions, work areas and other socio-demographic factors related to impacts of social isolation of university staff and students. The level of Significance was set at 0.05.

## Results

### Gender, age, location, role and scientific area of respondents

Of the 711 responses, the larger proportion consists of students (472), either undergraduate or postgraduate (67%), and females (64%) whose ages are between 20 and 40 years old (66%), and in the age band 21–30 (Table 4). Of the 238 academic staff, there was an even distribution across two age bands (66 responses in each) and a slightly higher response rate (69) in the 51–60 age band. Over 80% of respondents live in cities, about 12% in villages, and the remainder in rural areas. Some 27% live with a partner and another 18% live with both partner and children, while 22% live with parents. A smaller group lives either alone (10%), shares accommodation (10%), or lives with other relatives (8%), while a fraction lives with parents and partner (3%) or with children only (3%). (Appendix, Table 4*: Gender, age, and role of respondents).*

Most of the respondents worked or studied in European universities (83%). South American, North American, Central American, and African universities were represented by 1 to 14% of the respondents, and Australasian respondents by 3% (Table 5). More than 80% of the respondents worked or studied in the Sciences (social and physical), Engineering, Health Sciences, and Humanities/Linguistics. There is a comparatively higher concentration of respondents located in five countries: students and academic staff in German and Serbian institutions, academic staff in Brazilian and American Universities, and students in Portuguese universities. (Appendix, Table 5*: Country, role, and scientific area of respondents).*

### Duration respondents unable to work or study on campus

**Fig. 2:**
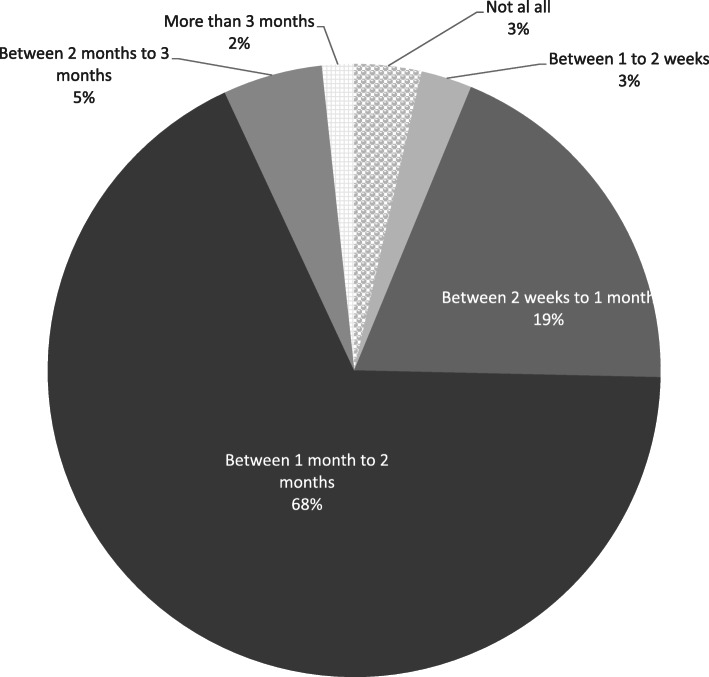
Duration respondents unable to work or study on campus

Some 5% of the respondents had been unable to work or study at their institution for 2 to 3 months, 68% for 1 to 2 months, 19% for up to 1 month, and only 3% of respondents had experienced no impact at all (Fig. 2).

### Extent to which respondents agree that their institutions shutdown operations

**Fig. 3:**
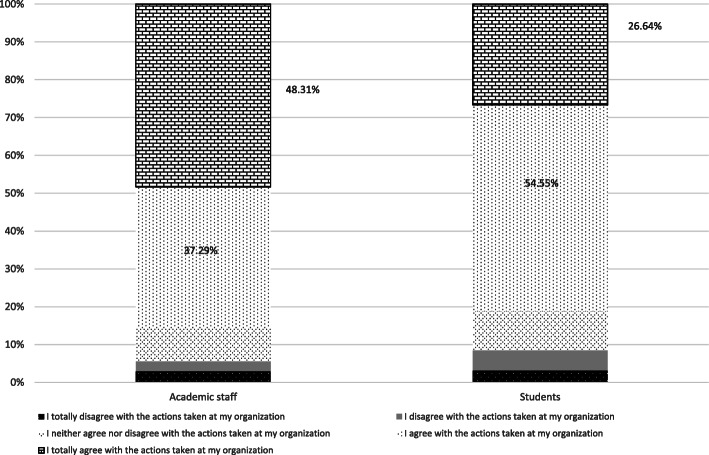
Extent to which respondents agree with their institution’s shutdown

Although more than 80% of the respondents agreed with the actions taken by their institutions, a significantly higher number of academic staff strongly agreed (48.31%), (academic staff: M = 4.25, SD = 0.94; students: M = 3.96, SD = 0.93; t(708) = 3.958, *p* < 0.05). More than half of students (54.4%)had no particular preference (Fig. 3).

### Country differences concerning attitudes to institutional shutdown

**Fig. 4:**
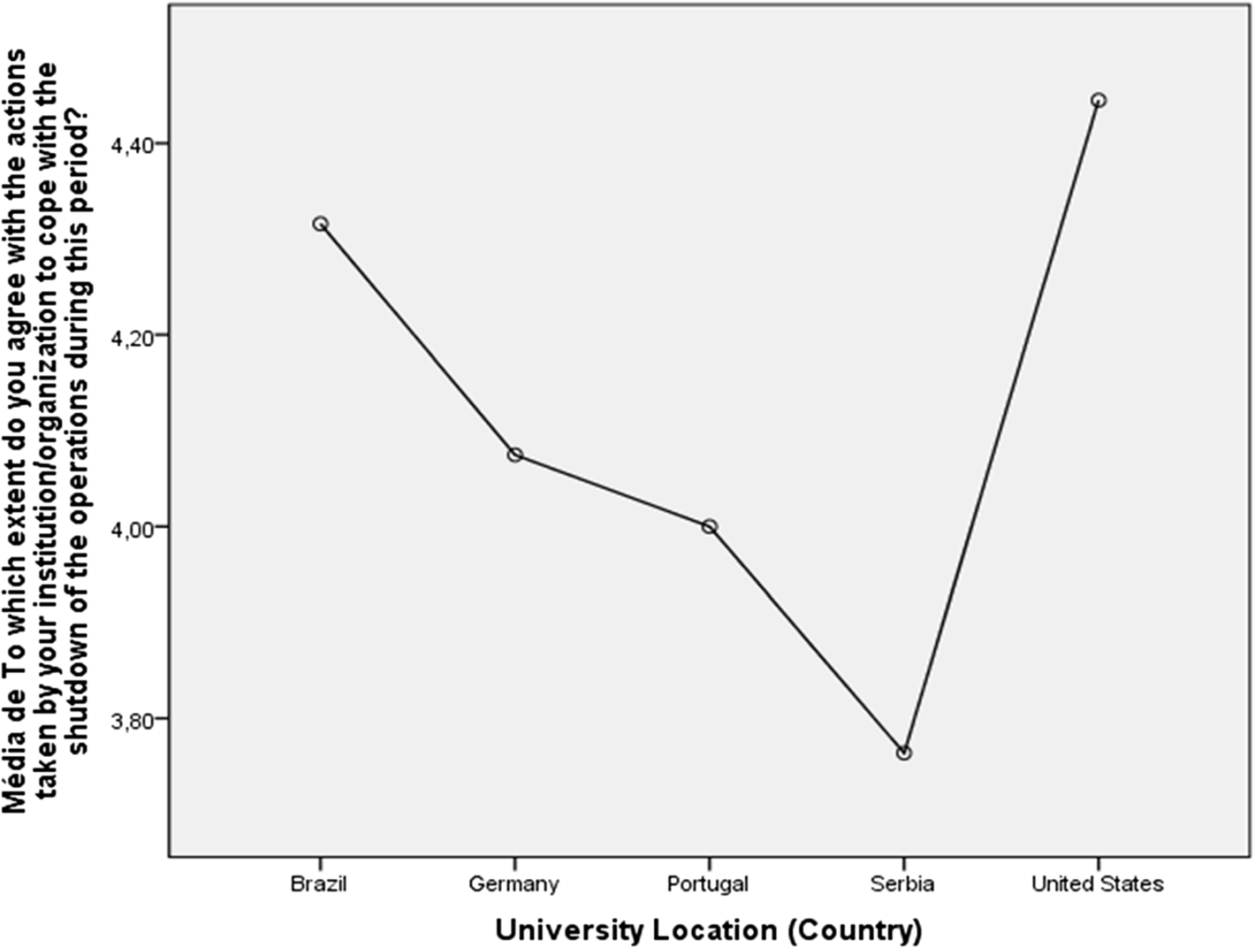
Country differences concerning attitudes to institutional shutdown

There is significant variation in how respondents of particular countries saw the need for institutional shutdown (Fig. 4). A one-way ANOVA test was conducted to compare the effect of geographical location (country) in the respondent’s attitude to their institution shutting down. There is a statistically significant difference amongst the five biggest country samples (F(4) = 5.496, *p* < 0.001). Respondents from the USA more strongly agreed with actions of their HEIs, while respondents in Serbia agreed the least.

### Working pattern of respondents

During the 2020 period of the pandemic crisis more than 90% of academic staff and 78% of students had worked from the ‘home office’. However, a small percentage of respondents stopped working altogether, especially students (15%) (Appendix, Table 6*: Working pattern of respondents).*

### Respondent evaluation of communications infrastructure while home working

**Fig. 5:**
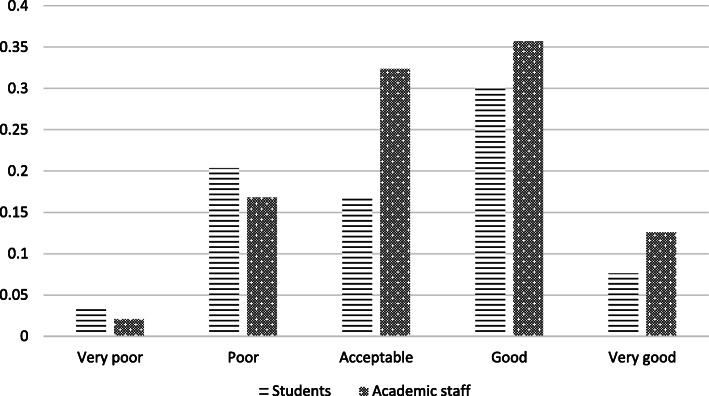
Respondent evaluation of communication infrastructure while home-working

Apart from phone use and emails during the shutdown, the most commonly used tools for communication included a wide variety of freely available platforms, the most common being Zoom (72,37%), followed by Microsoft Teams (45,76%) and Skype (39,57%), independently of whether or not the University had a virtual learning environment (VLE) platform. Academic staff showed significantly higher levels of satisfaction (academic staff: *M* = 3.40, *SD* = 0.98) compared with students: (*M* = 3.18, *SD* = 0.96); *t*(708) = 2.885, *p* < 0.05) regarding the performance of the communications infrastructure available at home. The mean value of both groups is above the midpoint of the scale (3) from 1 to 5 (Fig. 5).

### Respondent evaluation of HEI support for home working

**Fig. 6:**
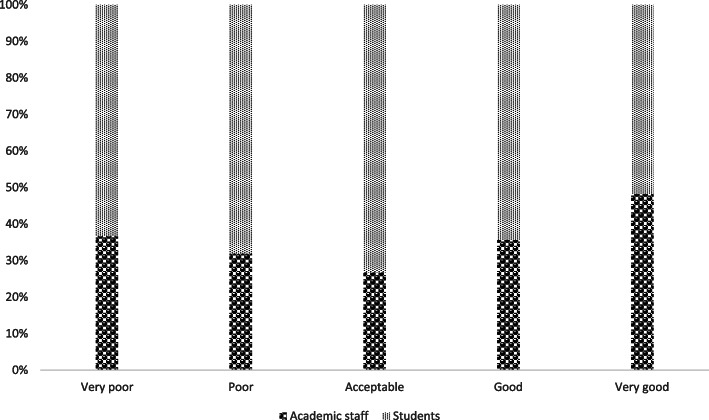
Respondent evaluation of HEI support for home-working

Mean values of both groups were above the midpoint of the scale (3) (Fig. 6). Nevertheless, academic staff seem significantly more satisfied with the support given by their university during the shutdown (academic staff: *M* = 3.40, *SD* = 0.73; students: *M* = 3.22, *SD* = 0.46, *t*(708) = 2.183, *p* < 0.05.

### Country effects on domestic infrastructure and institutional support

**Fig. 7:**
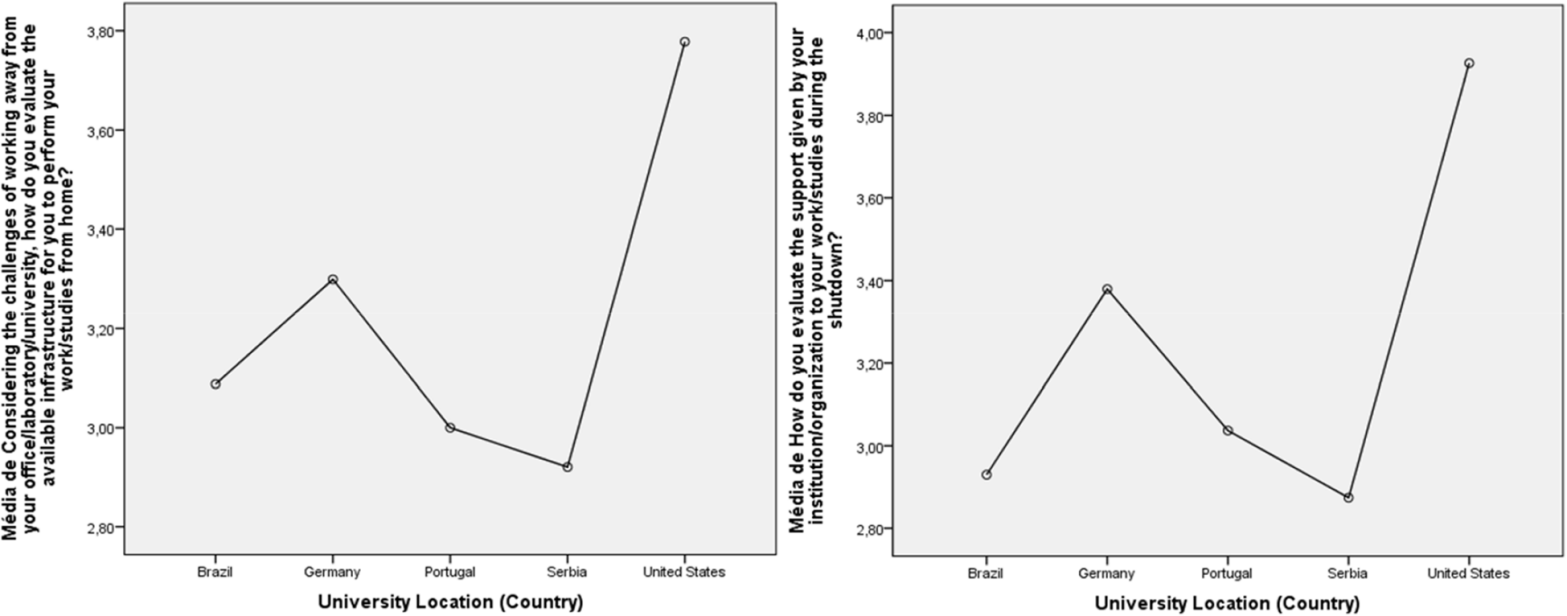
Perceived country differences on working or studying at home, in terms of: a) available infrastructure and b) institutional support

Using a one-way ANOVA test we compared: a) the effect of geographical location (country) o n the respondents’ evaluation of available infrastructure to work or study at home and b) the perceived support given by the institution during the shutdown. There is a statistically significant difference between the five biggest samples (Fig. 7). Respondents in the USA show a higher regard for their domestic infrastructure (3.80) as well as for the quality of their i nstitution’s support (4.00), compared to the other four country respondents.

The graph in Fig. 7 is to some extent consistent with that of Fig. 4, in that respondents based in the USA agreed with the shutdown, and are comparatively satisfied with their domestic technological communications infrastructure and, in the case of staff, with the support of their employing institution. At the other end of the spectrum, respondents in Serbia are least in agreement with the shutdown, least satisfied with their domestic communications infrastructure, and least satisfied with the support provided by their employing institution. Respondents in Brazil agree with the need for shutdown, while feeling significantly dissatisfied with their home-working communications infrastructure, and even less satisfied with their employing institution’s support for home working.

### Extent to which the shutdown has affected work or study

**Fig. 8:**
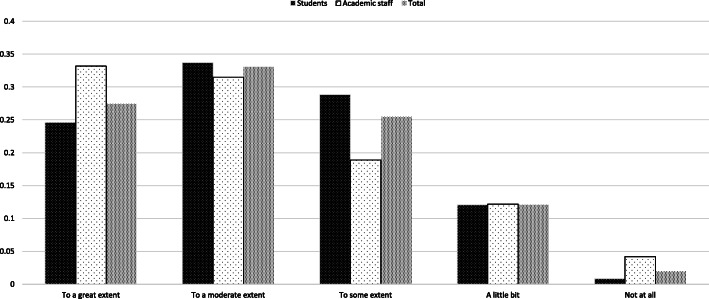
Extent to which shutdown has affected work or study

Both academic staff and students (more than 60% of the respondents) showed either ‘great’ or ‘moderate’ agreement, with a further 20% feeling ‘to some extent’ that the shutdown affected their work or study. (students: M = 2.31, SD = 1; academic staff: M = 2.23, SD = 1.16; t(708) = − 0.982, *p* > 0.05) (Fig. 8). There is clear unison in the perceptions of academic staff and students that the shutdown affected their work or study. Still, as Table 6*: Working pattern of respondents* shows, staff continued to work with little loss of momentum.

### Major problems experienced in work or study during the shutdown

**Fig. 9:**
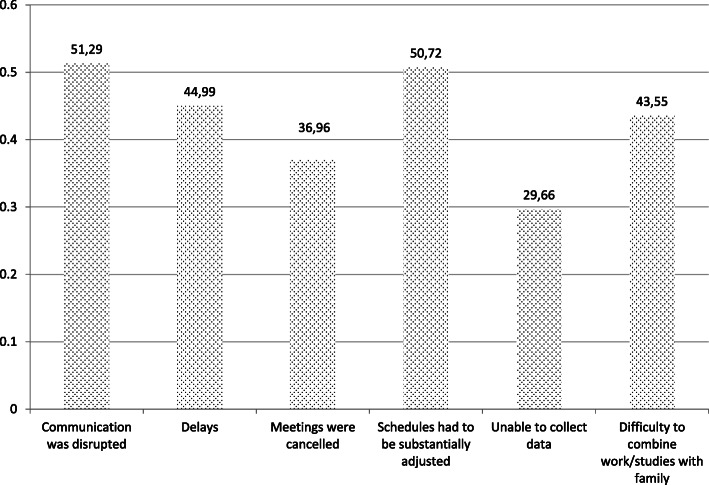
Major problems experienced in work or studies during the shutdown

The main functional problems that the respondents reported are: disruption of communication (51,29%) the adjustment of schedules (50,72%), delays (44,99%), the difficulty to combine work or studies with family (43,55%), the cancellation of meetings (36,96%), and the difficulty in collecting research data (29,66%) (Fig. 9).

Other problems mentioned (by approx. 30% of respondents) highlight not just functional problems but functional challenges overlaid with stress and anxiety. These include (a) feeling a lack of institutional support, (b) lacking motivation, (c) feeling the stress of living and working at home (as noted only around 10% live alone), (d) physical discomfort of working with unsuitable facilities at home and, for students e) the perception that professors were not willing or able to use online platforms.

### Impact of shutdown on workload

**Fig. 10:**
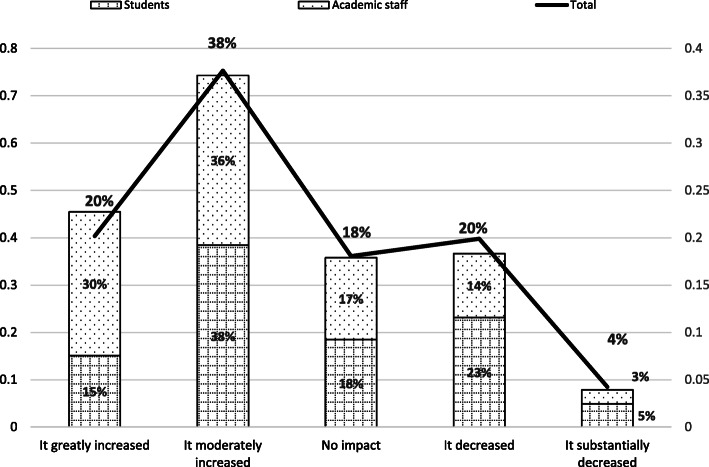
Impact of shutdown on workload

Almost 60% of respondents considered the shutdown as having a ‘moderate’ to a ‘greatly increased’ impact on their workload, while perhaps surprisingly, 20% indicated ‘no impact at all’. (Fig. 10). The difference between academic staff and student perceptions of workload increase is not significant (students: M = 2.64, SD = 1.13; academic staff: M = 2.23, SD = 1.11; t(706) = − 4.659, *p* > 0.05). A substantially higher proportion of students perceive a ‘decreased’ or ‘substantially decreased’ workload, which seems consistent with results reported in Table 6*: Working pattern of respondents*.

### Perceived impact of additional time indoors with family or roommates, during the shutdown

**Fig. 11:**
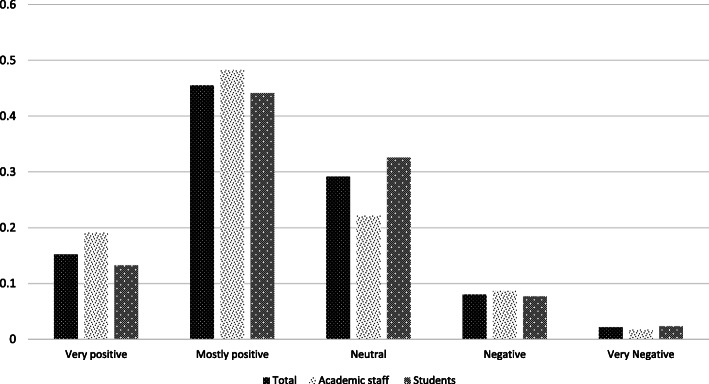
Perceived impact of confinement with family, roommates or colleagues, during the shutdown

As the above results show, most respondents feel the shutdown has negatively affected their work or study. At the same time the resulting confinement with family, roommates or friends (Fig. 11) is considered ‘positive’ and ‘mostly positive’ by more than 60% of respondents. Academic staff and students responded similarly.

### Religious commitments and attitudes to social confinement

**Fig. 12:**
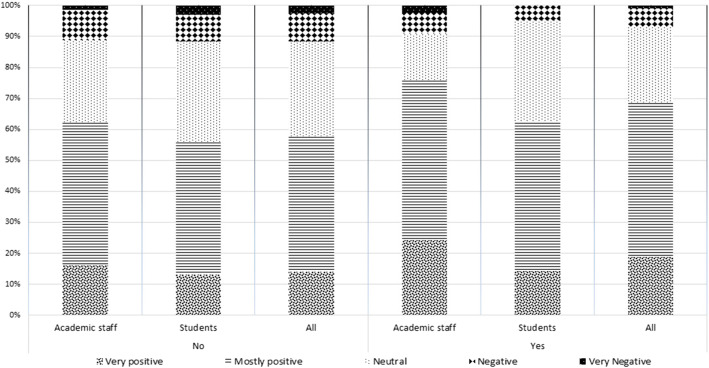
Religious commitments and attitudes to social confinement

Religious respondents (about 25% of respondents) fe lt comparably more positive about the confinement at home, with academic staff feeling the more positive (75%) (Fig. 12). The difference between non-religious and religious responses is statistically significant (t(690) = − 2.981, *p* < 0.05).

### Main challenges of social isolation due to COVID-19

**Fig. 13:**
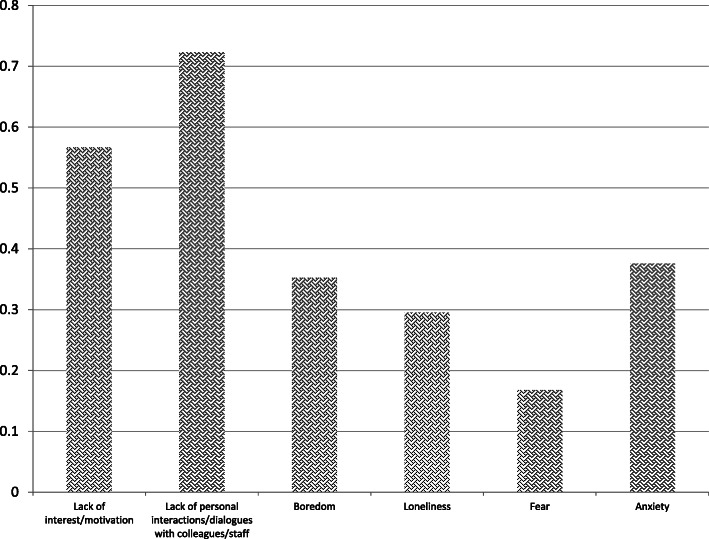
Main challenges of social isolation due to COVID-19

A substantial majority (70%) felt that the lockdown has adversely affected their work or study. The respondents noted that the main personal challenges due to the mandated social isolation (and not mutually exclusive) are: a lack of personal interactions with colleagues and staff (72%), a lack of motivation (57%), anxiety, and closely followed by boredom and loneliness (Fig. 13).

## Discussion

The vast majority of respondents considered that the shutdown had an adverse impact on their work, creating more of it, confirming that the shutdown disrupted their daily routines, especially with the ability to communicate with others and having to reschedule work and meetings. This is in line with results from Cao et al. [11] with college students in China. At the same time, respondents appreciated this time period as a unique opportunity to be with family. Academic staff were substantially more satisfied than students with their home-working infrastructure (e.g. ICT) and with their institutional support (see *Respondent evaluation of communications infrastructure while home working during the shutdown,* Fig. 5). Not surprisingly, and consistent with Cottini and Lucifora’s [44] observations, country differences do affect the pattern of response among respondents in terms of the nature of national HEI provision and support and the suitability of home-working arrangements, including information technology provision and communication infrastructure. In the USA, the respondents agree with their HEI’s shutdown decision, and have been satisfied with their home-working arrangements. At the other end of the spectrum, respondents in Serbia did see a need for their HEIs to shut down, but were not satisfied with their home-working arrangements.

Reflecting on *Working pattern of respondents* (Table 5), since the shutdown began during the 2019–2020 spring semester, academic staff and students in principle could have continued working, albeit communicating online and working independently from home. Yet may students report that they stopped working. One reason may be that while students had assessment work to complete, academic staff had assessment grading, along with administrative and on-going research work.

Consistent with other studies noted [26–28], this study shows clear evidence that the comprehensive shutdown of higher education – lasting three months (at the point of submitting this article) - had a detrimental impact on the mental health of a large proportion of academic staff and students (as noted some 70% feel adversely affected). The suddenly imposed social isolation led to staff and students experiencing problems of lack of social interactions, motivation, and mental health problems such as boredom, loneliness and anxiety (Fig. 13). These results confirm Cao et al.’s [11] correlation analysis that indicated varying levels of anxiety as being positively associated with numerous impacts (economic, on daily life, and delays in academic activities). Our findings, similar to Cao et al., indicate that anxiety results from stress caused by social isolation. Moreover, these *emotional* stresses overlay and amplify *functional* stresses, in the form of on-going work pressures (e.g. work load and academic deadlines) mixed with new stressors (sudden disruptive change to home-working, online working, and feeling that institutional support is inadequate). Yet another layer of anxiety among both academic staff and students is generated by the widely reported prospect of unemployment among academic staff, associated with the financial impacts on universities [15–17] and concerns about study programmes being interrupted or discontinued [11] a By way of textual evidence, the institution of one of the authors implemented redundancies, to which academics responded by consulting their union. Also, stress and anxiety levels are high among many doctoral students who were not able to work at home nor allowed access to the university buildings, a situation which impedes their progress which in turn has funding and completion implications. Our study results suggest a positive relationship between having a religious commitment and feeling positive about spending an unusually long period (weeks) at home with family. These results seem consistent with Chan et al. [46], whose longitudinal study shows that religious belief provides a sense of purpose for socially isolated individuals. The results are also consistent with Lauder et al.’s [47] survey of a random sample (of 1289 adults), which showed loneliness to be more prevalent among those with no religious belief. An earlier study by Tobacyk [48] of college students (average age 20 years) found no evidence of a relationship between traditional religious beliefs and a sense of alienation. Tobacyk [48] speculates that this might be because, as college students, this group lacks well-developed social-support systems such as ‘spiritualist colonies’. The results presented here show higher positive assessments of social isolation among both religious academic staff as well as students.

The considerable variation between countries of respondent attitudes to HEI shutdown, and in particular to their assessment of the adequacy of ICT infrastructure at home and HEI support, warrants closer examination. There may be many reasons for such differences, some widely reported, including the distinct rates of virus infection in individual countries, and the distinct speed and effectiveness of in-country leadership responses to the threat. Informing the latter are political judgements that rely (at least in part) on track and trace strategy and ICT infrastructure. Further, the variation in respondent assessments of home-working ICT infrastructure and HEI support, reported here, highlights the uneven scope and quality of HE institutional resources and preparedness for providing support (technical, organisational, emotional).

Since COVID-19 is an ongoing, global, public health emergency and given the potential for future epidemics, attention to the concerns for psychological health and wellbeing of staff and students, will require more attention from than HE sector policy makers and HEI leaders have to date given them. h As Wigginton et al. [49] urge, ‘academic institutions, governments, and funding agencies [must] develop practices and policies that encourage a more resilient, nimble, and equitable research ecosystem’. Our findings establish the need for this to be extended to include the HE sector more generally, including its teaching ecosystem.

As pandemic continues, bodies such as the American Council on Education and Universities UK may update their guidances, to incorporate ways of embracing online support services, the use of which could improve the quality and effectiveness of not only emergency interventions, but also as part of mainstream educational provision. For example, Liu et al. [50] reports on the development of Chinese public emergency interventions and the use of online mental health services in dealing with the COVID-19 epidemic. In their study of the Italian experience, D'Agostino et al. [51] agree with the value of greater investment in online health services.

Yet other responses are already emerging, as illustrated by another of the co-authors, whose UK university has responded by establishing a series of online services offered to staff and students that both nurture connectedness and reduce anxieties through, for example, collective mindfulness classes. The pandemic has revealed the vulnerability of the traditional HEI model of funding, based on the physical use of its estate for teaching and other peripheral services (e.g. accommodation and conferencing). Attempts to return to this model in the medium term is fraught with risk to health, yet many HEIs will feel compelled to take that risk in order to secure financial survival. HEIs, with the support of their governments, should be reflecting on existing practices and their recent experiences of (forced) online teaching and learning and encouraging research on teaching and learning, with the aim of evolving away from the *status quo* and developing new models of funding less reliant on physical consumption. This is an opportunity to cement what is being learnt about online teaching and learning (Lederman [52].

More broadly, the pandemic has laid bare the fault lines in the existing narrow formulation of HE as an enabler of economic growth through knowledge transfer or, as is conceptualised by Etzkowitz and Zhou’s [53], as just part of the triple helix ‘university-government-industry’. Rather, as Peter Wells, UNESCO’s Chief of the Higher Education Sector, observed years before the pandemic, ‘perhaps never before in recent history has the role of higher education been so intricately tied to the economic, social and environmental fabric of the modern world’ ([54]: 231).

The rippling impact of COVID-19 goes well beyond the internal machinations of HEIs or adjusting the ways academic staff and students interact. There is a new normal emerging, where teaching, learning and knowledge creation are unfolding in the context of social interactions (itself being reshaped) rather than in organisational contexts. As these new ways of working persist, civic society, policy makers, and HE practitioners need to reimagine how educational strategies might better support equality, the creation of knowledge, and the search for innovative ways of democratising work patterns and modes of learning, without the social cost of isolation. These seemingly divergent demands call for a broader integration of the university’s role within society, in turn requiring substantial changes to the existing HE ecosystem. Such integration should redress the over-reliance on HEI competition. Significant costs accompany the disciplining benefits of operating in a competitive market. As Mintz [55] reports, higher education in the USA (and no doubt in most market economies) is among the most stratified sectors in society, in terms of the individual HE institutional financial endowment, the capacity to offer financial aid to socially disadvantaged students, teaching and research budgets, and the capacity to prepare students post-graduation.

Exploring and renewing our understanding of higher education within society becomes a new research agenda. Drawing on Cai [56], creating and maintaining sustainable societies as envisioned here requires integrating the idea of a university within a civil society ecology that is seen as a complex system, with emergent and resilient properties constituted of continually negotiated interactions between the university, civil society governing bodies, and competing interests (economic, social, and environmental).

### Policy implications

Having analysed the problems, difficulties and constraints caused by and/or associated with social isolation, it is now important to look ahead. There are some measures that may be deployed in the future in order to allow staff and students to better cope with social isolation in cases of new or recurring pandemics. These are:

- The provision of psychological care and support to academic staff in order to better equip them to cope with the additional burdens of home schooling on the one hand, and meeting teaching schedules on the other;
- The provision of counselling to students, in order to reduce the anxiety caused by social isolation and foster a better work-life balance;
- A greater use of online activities (including religious services and cultural events). Many organizations offer digital gatherings of all sorts, which may be used as a means of getting in touch with more people;
- The set-up of informal communication channels in order to facilitate and encourage conversations in both groups, which helps people to feel less alone and more supported.

More items may be added to the list, but the above are examples of what can be done in a rather simple way and without major costs or investments.

As a complement to the above measures, a review of content delivery and the ways lectures are organized and held should also be performed. The psychological pressures that staff and students are exposed to means that traditional teaching - and evaluation – models are not suitable. Rather, academic staff needs to consider innovative ways of communicating study contents to students, in a way that takes into account the many concerns and worries they have and the pressures they are subjected to, as a result of social isolation.

Despite its scope, this paper has some limitations. The first is the size of the sample, which entails 711 responses. In addition, the number of countries investigated, namely 41, cannot be regarded as representative of the world. Also, the responses varied among countries, meaning that some countries had more respondents than others. Moreover, the fact that the study looked at the attitudes of academic staff and students means that a disaggregation from their opinions is not always possible. Apart from the fact that various other studies are currently being undertaken which look at either group, the rationale behind the approach used here is that the authors wanted to offer an overall picture of the extent to which these two major groups (academic staff and students) are being affected.

But despite these constraints, the study is a welcome addition to science in the sense that it offers an overview of the many aspects associated with social isolation in academic life and illustrates its impacts on academic staff and students. Also, the sampling, which involved 41 countries, allows one to build a rough international profile of the impacts of social isolation in a university context.

## Conclusions

This paper has analyzed the impacts of the lockdowns triggered by the COVID-19 pandemic on academic life, identifying the extent to which the universities´ operations were disturbed and paying special attention to aspects related to social isolation.

It is evident that there were significant differences in the reactions to the lockdowns by academic staff and students. Whereas most students stopped working immediately after the lockdowns, most academic staff continued to work. In addition, academic staff showed a greater level of satisfaction with their provisions and facilities for working during the special situation caused by the pandemic, whereas students indicated they were not satisfied. In light of the growing awareness of ‘digital poverty’ and the ‘digital divide’ which define students’ absolute and relative access to IT equipment and internet, an obvious reason may be that they were ill equipped to cope with the sudden change to on-line learning.

There are also differences in the degree of influence of some of the problems, when compared between countries.. For instance, students in the United States considered that their institution’s infrastructure was in better shape to cope with the lockdowns, when compared with those from the other sampled countries.

As the world finds itself in the middle of a second wave by the time this paper has been written, it is clear that universities need to be mindful of the many impacts the pandemic will have in their operations, at present and in the future.

Finally, one item that also deserves mentioning is that people have been required to remain home and avoid contact with third parties; this means that an opportunity is given for quality family time. A greater understanding of the impacts of social isolation and of some of the means by which its impacts may be mitigated, as this paper has tried to outline, may lead to a better preparedness of academic staff and students for handling such events now that pandemics are realities on our collective future horizons.

### Supplementary Information

**Additional file 1.** Questionairre.

## Data Availability

The datasets during and/or analysed during the current study available from the corresponding author on reasonable request.

## Appendix

**Table 3:** Summary of questionnaire design

*Demographic data:* Q1-Q6, Q.17 (live with whom) & Q18 (urban or rural area)	
*Impact on work practices (functional):*Q7-Q15	*Impact on wellbeing (emotional):* Q16, Q19, Q20/21, Q22
Q7- length of isolation Q8 - agree with shutdown decision? (spill over to emotional) Q9 - work/study normal, home-working, both, no work Q10- except email, which comms technology used Q11 - quality of infrastructure: very poor to very good Q12 - quality of HE support very poor to very good Q13 - affected work (effort/performance): more/less Q14 - problems or home-working: comms, delays, others Q15 - impact of shutdown on workload: more/less work Q17 - live alone or with others	Q16 - impact on work/study: (spill-over from functional) Q19 - forced confinement with others: positive vs negative Q20 - Religious? Y or N Q21 - if Y to 20, is it comforting? Q22 – main challenges: loneliness, boredom, others

**Table 4:** Gender, age, and role of respondents

Gender and age	Academic staff	Students	n.a.	Total
**Total**	**238**	**472**	**1**	**711**
Up to 20 years old	0	73	/	73
21 to 30 years old	15	344	/	359
31 to 40 years old	66	44	/	110
41 to 50 years old	66	9	/	75
51 to 60 years old	69	2	/	71
More than 60 years old	22	/	/	23
**Female**	**147**	**309**	**/**	**456**
Up to 20 years old	/	51	/	51
21 to 30 years old	10	218	/	228
31 to 40 years old	49	31	/	80
41 to 50 years old	37	8	/	45
51 to 60 years old	44	1	/	45
More than 60 years old	7	/	/	7
**Male**	**91**	**163**	**1**	**255**
Up to 20 years old	/	22	/	22
21 to 30 years old	5	126	/	131
31 to 40 years old	17	13	/	30
41 to 50 years old	29	1	/	30
51 to 60 years old	25	1	/	26
More than 60 years old	15	/	1	16

*n.a* not applicable

**Table 5:** Country, role, and scientific area of respondents

	Scientific Area							
	Humanities/Linguistics	Social Sciences	Sciences^a^	Health Sciences	Engineering	Other	n.a.	Total
**Role**								
**Academic staff**	**11**	**82**	**24**	**32**	**48**	**41**	**/**	**238**
Brazil	3	10	5	15	7	1	/	41
Germany	1	8	7	13	18	11	/	58
Portugal	/	3	2	1	1	3	/	10
Serbia	1	12	1	/	14	2	/	29
USA	1	12	1	/	1	4	/	19
Other	5	37	7	2	7	20	/	78
n.a.	/	/	1	1	/	1	/	3
**Student**	**11**	**34**	**47**	**113**	**196**	**71**	**1**	**472**
Brazil	1	2	5	5	1	2	/	16
Germany	1	5	25	100	123	49	/	303
Portugal	/	2	2	4	/	9	/	17
Serbia	7	8	6	1	70	3	1	96
USA	/	6	1	1	/	/	/	8
Other	2	11	8	2	2	7	/	32
n.a.	/	/	/	/	/	1	/	1
**Total**	**22**	**28**	**71**	**145**	**244**	**112**	**1**	**711**

*n.a* not applicable

^a^Biological, Chemical, Physical, Earth, Agrarian, Mathematical by descending order of number of respondents

**Table 6:** Working pattern of respondents

Role	Working normally from office/laboratory	Working from “home office” only	Moving regularly between home and office/laboratory	Have stopped working (no activities/University full shutdown)
Academic staff	2%	91%	6%	1%
Student	3%	78%	4%	15%
Total	3%	82%	5%	10%

## Abbreviations

HE: Higher education
HEIs: Higher education institutions
ICT: Information and communications technology
UN DESA: The United Nations department of economic and social affairs
UNESCO: The United Nations educational, scientific and cultural organization
WHO: World health organization
COVID-19: Corona virus disease 2019
SARS-CoV-2: Severe acute respiratory syndrome coronavirus 2
SPSS: Statistical package for the social sciences

## Footnotes

1 Argentina, Australia, Austria, Belgium, Brazil, Bosnia and Hercegovina, Bhutan, Germany, Canada, Colombia, England, Estonia, Finland, Greece, Guatemala, India, Indonesia, Iraq, Israel, Italy, Kenya, Kyrgyzstan, Malta, Mauritius, Mexico, New Zealand, Nigeria, Norway, Poland, Portugal, Romania, Scotland, Serbia, Slovenia, South Africa, Spain, Sri Lanka, Sweden, Switzerland, Ukraine, United States of America (USA)
