# Supplementary material for: Impacts of COVID-19 and social isolation on academic staff and students at universities: a cross-sectional study
Source: BMC Public Health. 2021 Jun 24;21:1213. doi: 10.1186/s12889-021-11040-z (PMC8223197; doi:10.1186/s12889-021-11040-z)
Supplement: Supplementary file 1 — Additional file 1. Questionairre. [file 12889_2021_11040_MOESM1_ESM.docx]

| **COVID-19 and Social Isolation among university staff and students**  **A study undertaken by the Inter-University Sustainable Development Research Programme (IUSDRP) & the European School of Sustainability Science and Research (ESSSR) - 0** |
| --- |

This questionnaire is part of a study that intends to understand the impacts of social isolation among university staff and students at universities round the world, as a result of the crises caused by COVID-19.

Your participation is voluntary and there are no right or wrong answers. We are interested in knowing your personal understanding.

Please answer all questions by checking the answer (s) that best suit your opinion. Whenever you select the "Other" answer option, please state which ones you refer to by writing in the space reserved for that purpose.

It should be noted that the answers given in this questionnaire are strictly confidential, is used only for statistical treatment. We commit to respect the protection of personal data, guaranteeing its confidentiality.


Thank you in advance for your cooperation. Your participation is essential!

Please let Aprajita Minhas know, if you wish to receive the results of the study: Aprajita.Minhas@haw-hamburg.de.

Top of Form

**1. My  role at University**

Member of staff  (eg. lecturer, professor)

Student (undergraduate/postgraduate

**2. Gender**

Male

Female

**3. University Location (Country)**

**4. University Name**

**5. Age**

up to 20 years old

21 to 30 years old

31 to 40 years old

41  to 50 years old

51  to 60 years old

more than 60 years old

**6. In which Scientific area do you work?**

Earth Sciences

Biological Sciences

Chemical Sciences

Physical Sciences

Mathematical Sciences

Engineering

Health Sciences

Agrarian Sciences

Social Sciences

Humanities/Linguistics

Other (please specify)

**7. How long have you been affected by the shutdown and unable to perform normal work /studies at your institution?**

Not at all

Between 1 to 2 weeks

Between 2 weeks to 1 month

Between 1 month to 2 months

Between 2 months to 3 months

More than 3 months

**8. To which extent do you agree with the actions taken by your institution/organization to cope with the shutdown of the operations during this period?**

I totally disagree with the actions taken at my organization

I disagree with the actions taken at my organization

I neither agree nor disagree with the actions taken at my organization

I agree with the actions taken at my organization

I totally agree with the actions taken at my organization

**9. During this crisis, you:**

are working normally from your office/laboratory

are working at “home office” only

are shutting regularly between home and your office/laboratory

have stopped working (no activities/university full shutdown)

**10. Apart from e-mails, which tools have you used for communication during the shutdown? Multiple answers possible**

Zoom

Skype

Webex

Microsoft Teams

Other (please specify)

**11. Considering the challenges of working away from your office/laboratory/university, how do you evaluate the available infrastructure for you to perform your work/studies from home?**

Very poor

Poor

Acceptable

Good

Very good

**12. How do you evaluate the support given by your institution/organization to your work/studies during the shutdown?**

Very poor

Poor

Acceptable

Good

Very good

**13. To which extent has the shutdown influenced your work/studies?**

To a great extent

To a moderate extent

To some extent

A little bit

Not at all

**14. During the shutdown, which problems have you experienced in your work/studies? (Multiple answers possible)**

Communication was disrupted

Delays

Meetings were cancelled

Schedules had to be substantially adjusted

Unable to collect data

Difficulty to combine work/studies with family

Other (please specify)

**15. How do you rate the impacts of the COVID-19 crisis on your workload?**

It greatly increased

It moderately increased

No impact

It decreased

It substantially decreased

Please explain

**16. How do you evaluate the impact of the social isolation caused by COVID-19 on your work/studies?**

It has helped to improve my work/studies

It has negatively impacted my work/studies

Please explain your answer

**17. Please tick the condition which better describe how you live:**

Alone, in a single household

With a spouse/ partner

With my partner and children

With my children alone

With my partner and parents

With my parents

With relatives or other family members

With roommates

With fellow university colleagues

**18. Please tick the condition which better describe how you live:**

in a city centre

in city outskirts

in a village

in the countryside

**19. How do you rate the additional time indoors with your family, roommates, colleagues or relatives during the shutdown?**

Very positive

Mostly positive

Neutral

Negative

Very Negative

**20. Do you practice any religion or are a member of any confession?**

Yes

No

**21.  If Yes, has your religion or confession played a role (e.g. comforting, moral and spiritual support) during the shutdown?**

Yes, quite significantly

Yes, to a great extent

Yes, to some extent

Yes, a little bit

Yes, but very little

**22. Which are/were the main challenges of social exclusion due to COVID-19? (Multiple answers possible)**

Lack of interest/motivation

Lack of personal interactions/dialogues with colleagues/staff

Boredom

Loneliness

Fear

Anxiety

**23. To what extent has the shutdown affected your sense of:**

|  | Not at all | Somewhat | Not sure | Often | Very often |
| --- | --- | --- | --- | --- | --- |
| Optimism about the future |  |  |  |  |  |
| Personal Usefulness |  |  |  |  |  |
| Relaxation |  |  |  |  |  |
| Interest in other people |  |  |  |  |  |
| Your ability to deal with the problem |  |  |  |  |  |
| Your ability to think clearly |  |  |  |  |  |
| Feeling good about yourself |  |  |  |  |  |
| Closeness to other people |  |  |  |  |  |
| Confidence |  |  |  |  |  |
| Ability to make up your own mind about things |  |  |  |  |  |
| Feeling loved |  |  |  |  |  |
| Being interested in new things |  |  |  |  |  |
| Cheerfulness |  |  |  |  |  |
| Physical fitness |  |  |  |  |  |

**24. Has COVID-19 influenced you to adapt/change your lifestyle?**

Yes

No

If “Yes”, what form has this taken?

**25. To what extent did the shutdown affect physical exercise:**

I have done more exercise at home

I have done less exercise overall

**26. Will the COVID-19 shutdown and social isolation likely to continue to affect you in the long-term?**

Yes

No

Not sure

**27. If “YES”, in which ways:**

I will travel more

I will attend more physical events

I will make more use of IT-based communication technologies

I will prepare myself better for “home office”

I will spend more time with family and friends

I will work/study harder

Other (please specify)

**28. How do you think the COVID-19 shutdown will affect you in the long-term?**

|  | Not at all | Somewhat | Not sure | Often | Very often |
| --- | --- | --- | --- | --- | --- |
| Optimism about the future |  |  |  |  |  |
| Personal Usefulness |  |  |  |  |  |
| Relaxation |  |  |  |  |  |
| Interest in other people |  |  |  |  |  |
| Your ability to deal with the problem |  |  |  |  |  |
| Your ability to think clearly |  |  |  |  |  |
| Feeling good about yourself |  |  |  |  |  |
| Closeness to other people |  |  |  |  |  |
| Confidence |  |  |  |  |  |
| Ability to make up your own mind about things |  |  |  |  |  |
| Feeling loved |  |  |  |  |  |
| Being interested in new things |  |  |  |  |  |
| Cheerfulness |  |  |  |  |  |
| Physical fitness |  |  |  |  |  |

**29. If relevant, please indicate other aspects related to the impact of the COVID-19 shutdown that you consider important and that were not addressed in the previous questions.**
